# Neural substrates predicting short-term improvement of tinnitus loudness and distress after modified tinnitus retraining therapy

**DOI:** 10.1038/srep29140

**Published:** 2016-07-06

**Authors:** Shin Hye Kim, Ji Hye Jang, Sang-Yeon Lee, Jae Joon Han, Ja-Won Koo, Sven Vanneste, Dirk De Ridder, Jae-Jin Song

**Affiliations:** 1 Department of Otolaryngology-Head and Neck Surgery, Korea University College of Medicine, Seoul, Korea; 2 Department of Otorhinolaryngology-Head and Neck Surgery, Seoul National University Bundang Hospital, Seongnam, Korea; 3 School of Behavioral and Brain Sciences, The University of Texas at Dallas, United States of America; 4 Unit of Neurosurgery, Department of Surgical Sciences, Dunedin School of Medicine, University of Otago, Dunedin, New Zealand; 5 BRAI^2^N, Sint Augustinus Hospital, Antwerp, Belgium

## Abstract

Although tinnitus retraining therapy (TRT) is efficacious in most patients, the exact mechanism is unclear and no predictor of improvement is available. We correlated the extent of improvement with pre-TRT quantitative electroencephalography (qEEG) findings to identify neural predictors of improvement after TRT. Thirty-two patients with debilitating tinnitus were prospectively enrolled, and qEEG data were recorded before their initial TRT sessions. Three months later, these qEEG findings were correlated with the percentage improvements in the Tinnitus Handicap Inventory (THI) scores, and numeric rating scale (NRS) scores of tinnitus loudness and tinnitus perception. The THI score improvement was positively correlated with the pre-treatment activities of the left insula and the left rostral and pregenual anterior cingulate cortices (rACC/pgACC), which control parasympathetic activity. Additionally, the activities of the right auditory cortices and the parahippocampus, areas that generate tinnitus, negatively correlated with improvements in loudness. Improvements in the NRS scores of tinnitus perception correlated positively with the pre-TRT activities of the bilateral rACC/pgACC, areas suggested to form the core of the noise-canceling system. The current study supports both the classical neurophysiological and integrative models of tinnitus; our results serve as a milestone in the development of precision medicine in the context of TRT.

Tinnitus is described as the perception of sound or noise in the absence of any corresponding external acoustic stimulation. The overall prevalence of tinnitus in the adult population ranges from 10–15% across studies; 5% of the population has severe distressing tinnitus that can disrupt daily activities, resulting in a negative impact on the quality of life^1^,^2^. Various management strategies have been developed to help reduce tinnitus symptoms. Applied in conjunction with specific treatment for underlying or co-existing abnormalities, management strategies for tinnitus include tinnitus retraining therapy (TRT), cognitive behavioral therapy, sound therapy, hearing rehabilitation using hearing aids or cochlear implants, pharmacotherapy, and brain stimulation^3^.

Of these treatment modalities, TRT is a combination of directive counselling and sound therapy that seeks to manage tinnitus and reduce sound tolerance, based on a neurophysiological model of tinnitus^4^. A crucial feature of the model is that functional connections between the auditory, limbic, and autonomic nervous systems are considered to be responsible for symptoms such as anxiety, problems with concentration, and suppression of the ability to enjoy daily activities^4^,^5^,^6^. The first findings that supported this model were published in 1998^7^, and many subsequent studies have shown that various systems of the brain are involved in tinnitus^8^,^9^,^10^,^11^,^12^,^13^. The aim of TRT is to suppress tinnitus by extinguishing functional connections between the auditory, limbic, and autonomic nervous systems. This creates habituation of tinnitus-evoked reactions and ultimately the phantom perception per se^5^.

Over 100 studies have explored the effectiveness of TRT, and most publications have indicated that significant improvements in tinnitus were possible in about 80% of patients^5^. Although only two controlled clinical trials of TRT have been published, both studies showed that TRT effectively treated tinnitus^14^,^15^. In addition, in the time since TRT was first introduced, many modifications and improvements have been developed. For example, the average time to attainment of clear improvement was reduced from 1 year to 1 month, with a statistically significant improvement evident at 3 months^5^. Further, modified TRT using small-group directive counseling and ambient sound stimulation has proven to be effective for tinnitus relief^16^.

Neither the precise mechanism of tinnitus improvement nor predictors of the degree of improvement in tinnitus after TRT are fully understood. Recently, a revised integrative model of auditory phantom perception based on the classical neurophysiological model was suggested^17^. This recent model suggested that changes in the activities and functional connections between auditory areas, including the auditory cortices^18^,^19^,^20^, the parahippocampus^21^, and limbic/autonomic areas (the bilateral rostral/pregenual/subgenual anterior cingulate cortices^22^,^23^,^24^, the bilateral ventral medial prefrontal cortex^25^,^26^, and the bilateral insula), are important in the maintenance of tinnitus. TRT would be expected to change the connectivity between auditory and limbic brain structures and also between the auditory and autonomic nervous systems controlling various areas of the brain.

Most studies have reported 80% success rates; about 20% of patients do not benefit from TRT. In this regard, it is essential to explore possible pre-TRT predictors of tinnitus improvement. We correlated the extent of tinnitus improvement 3 months after the first TRT session with pre-TRT resting-state source-localized quantitative electroencephalography (rs-qEEG) findings. This was because we sought to identify the pretreatment neural substrates that afforded short-term tinnitus improvement after TRT. We also compared the qEEG findings of patients that experienced good outcomes after TRT with those who experienced poor outcomes. To this end, we used source localization complimented by connectivity analysis.

## Materials and Methods

### Participants

Thirty-two consecutive subjective tinnitus patients were prospectively enrolled in a TRT program at Seoul National University Bundang Hospital, and informed consent was obtained from all subjects (IRB-B-1604-344-302). The enrolled subjects’ mean Tinnitus Handicap Inventory (THI) score, a self-report tinnitus handicap measure comprising 25 items exploring the extent of tinnitus-related distress^27^, was 57.8 ± 20.7 (equal to “severe” handicap defined by Newman *et al*.)^28^. The mean age of the patients was 50.8 ± 15.2 years (range, 20–81 years), and 18 were males. At the initial visit, we obtained a structured medical history regarding the characteristics of each patient’s tinnitus, such as the affected side, the nature (pure-tone or narrow-band noise), and the symptom duration. Of the 32 patients, 16 complained of unilateral tinnitus while the other 16 had bilateral tinnitus. Twenty-four patients had pure-tone tinnitus, while the other 8 had narrow-band noise tinnitus. The mean symptom duration was 4.0 ± 5.1 years (range, 6 months–25 years).

All participants underwent pure-tone audiometry (PTA); psychoacoustic tests of tinnitus, such as tinnitus pitch matching, tinnitus loudness matching, and the minimum masking level test^29^; and an initial pre-TRT EEG. The perceived tinnitus handicap was measured using the THI^27^. We derived numeric rating scale (NRS) scores of tinnitus loudness (answering the question “how loud is your tinnitus?” on a scale from 0 to 10), tinnitus-related distress (answering the question “how bothered are you by your tinnitus?” on a scale from 0 to 10), and the percentage of the daytime during which the participant was aware of the tinnitus (NRS tinnitus perception, 0–100%). All tests were conducted both before and 3 months after the initial TRT session.

Individuals with hearing loss of more than 40 dB in at least one ear (calculated by averaging the PTA thresholds at 0.5, 1, 2, and 3 kHz) were excluded from the study^30^. However, 5 subjects with mild hearing loss (less than 30 dB) that did not necessitate hearing aid were included in the study. We also excluded patients with pulsatile tinnitus, Ménière’s disease, otosclerosis, psychiatric/neurological disorders, histories of drug or alcohol abuse, and chronic headache; those using current psychotropic or central nervous system-active medications; those with any history of head injury involving loss of consciousness; and those with a history of seizures. The study was approved by the Seoul National University Bundang Hospital Institutional Review Board and was conducted in accordance with the Declaration of Helsinki.

### The modified TRT program

After the initial evaluation, all subjects participated in a TRT session consisting of directive counseling based on the neurophysiological model of Jastreboff 6. For approximately 40 min, the initial TRT session covered the following content: the definition and incidence of tinnitus, the simplified anatomy of the ear and the auditory pathways, the subconscious processing and conscious perception of auditory stimuli, selective listening and the central noise-canceling mechanism^9^, the vicious circle of listening and reacting, the neurophysiological model, tinnitus demystification, motivation and reassurance, an explanation of habituation and ignorance of tinnitus as the goal of TRT, the roles played by hearing aids and various sound therapies in treating tinnitus, and advice on how to avoid silence and to add non-noxious sounds to the daily environment (i.e., ambient sound stimulation). All subjects were recommended to add ambient sound stimulation (e.g., radio, television, or background music) at least 6 hours every day while they are awake not to be exposed to complete silence. Hearing aid was not used in the current study as only patients with normal hearing or mild hearing loss were included. After the initial TRT session, all subjects were scheduled for monthly follow-up TRT sessions. At each follow-up session, all subjects were re-evaluated using follow-up questionnaires and were counseled for about 30 min to determine subjective changes in their symptoms and to review the core content of the initial counseling session.

### EEG recording

The EEGs were recorded for 5 min using a tin electrode cap (Electro-Cap, Ohio, United States), a Mitsar amplifier (EEG-201; Mitsar, St. Petersburg, Russia), and WinEEG software version 2.84.44 (Mitsar) in a fully lit room shielded from sound and stray electric fields. The participants sat upright in a comfortable chair with their eyes closed. Impedances were maintained below 5 kΩ for all electrodes throughout the EEG recording. Data were recorded at a sampling rate of 1,024 Hz, high-pass filtered at 0.15 Hz, and low-pass filtered at 200 Hz. The raw data were initially processed by resampling to 128 Hz, and band-pass filtered using a fast Fourier transform filter and application of a Hanning window at 2–44 Hz. The data were imported into Eureka! software^31^, plotted, and carefully inspected; artifacts were removed manually. Specifically, we carefully removed all episodic artifacts, including eye movements, blinking, teeth clenching, and body movements, from the EEG stream.

Patients were instructed not to drink alcohol for 24 h prior to the EEG measurement to avoid alcohol-induced changes in the EEG^32^. They were also told to abstain from caffeinated beverages on the day of the EEG measurement to avoid caffeine-induced decreases in the alpha and beta powers^33^,^34^. Participant vigilance was checked by monitoring abnormal EEG streams (e.g., slowing of the alpha rhythm) or the appearance of spindles (i.e., to prevent possible enhancement of the theta power due to drowsiness)^35^. No participant exhibited such drowsiness-related EEG changes.

### Source localization analysis

Low-resolution brain electromagnetic tomography (LORETA)-KEY software, available at http://www.uzh.ch/keyinst/NewLORETA/Software/Software.htm, dedicated to functional localization of standardized current densities based on certain electrophysiological and neuroanatomical constraints^36^, was used to localize the cortical sources that generated the scalp-recorded electrical activities in each of the following eight frequency bands: delta (2–3.5 Hz), theta (4–7.5 Hz), alpha 1 (8–10 Hz), alpha 2 (10–12 Hz), beta 1 (13–18 Hz), beta 2 (18.5–21 Hz), beta 3 (21.5–30 Hz), and gamma (30.5–44 Hz)^8^,^9^,^10^,^11^,^13^,^37^. The standardized LORETA (sLORETA) algorithm solves the inverse problem, which is source reconstruction from electric neuronal activities based on extracranial measurements. The software makes certain assumptions in terms of the orientations and strengths of the neighboring neuronal sources represented by adjacent voxels.

The solution space used was a three-shell spherical head model registered to a standardized stereotactic space implemented in the LORETA-KEY software (available at http://www.uzh.ch/keyinst/loreta.htm). This software implements revisited realistic electrode coordinates^38^ and the lead field of Fuchs *et al*.^39^ to apply the boundary element method introduced by the Montreal Neurological Institute (MNI)-152 (Canada). The sLORETA-KEY anatomical template divides the neocortical MNI-152 volume, including the hippocampus and anterior cingulate cortex, into 6,239 voxels with dimensions of 5 × 5 × 5 mm, based on probabilities returned by the Daemon Atlas^40^. Anatomical labeling of significant clusters was performed automatically by a toolbox within sLORETA. The locations of significant clusters were initially determined using this toolbox^41^, and were reconfirmed by referencing the Talairach and Tournoux atlas^42^.

### Functional connectivity analysis

Phase synchronization and the extent of coherence between the time series corresponding to different regions of interest (ROIs) were calculated to analyze functional connectivity; we employed the built-in sLORETA connectivity toolbox to this end. This toolbox defines measures of linear- and non-linear dependence (i.e., coherence and phase synchronization) between multivariate time series; the measures are expressed as the sums of lagged/instantaneous dependencies. For functional connectivity analysis, a total of 28 ROIs defined by Brodmann area (BA) were selected as possible nodes based on previous literature on tinnitus: the bilateral primary and secondary auditory cortices^18^,^19^,^20^, the bilateral parahippocampus^21^, the bilateral rostral/pregenual/subgenual anterior cingulate cortices [ACCs]^22^,^23^,^24^, the bilateral ventral medial prefrontal cortex [vmPFC]^25^,^26^, and the bilateral insula.

### Statistical analysis

The statistical method used for source localization and connectivity analyses was Statistical Non-parametric Mapping (SnPM), employing permutation tests on labels as comparisons. The SnPM method handles with the multiple comparisons problem inherent in the standard voxel-by-voxel hypothesis-testing framework. SnPM yields results similar to those obtained by Statistical Parametric Mapping, but employs a general linear model featuring corrections for multiple comparisons; the model is based on random field theory. In brief, the SnPM corrects for the fact that multiple tests are performed on all voxels and all frequency bands. As the method is nonparametric in nature, the validity thereof does not rely on any Gaussian assumptions^43^.

In this way, we correlated pre-TRT source-localized activities among whole-brain areas defined by Brodmann (i.e., BAs). We defined connectivity as the percentage improvement in the THI score, NRS loudness, and NRS perception. We used the median split method^13^,^44^, a data-driven post-hoc stratification approach, to further test group differences between the marked improvement [MI] and slight improvement [SI] groups with regard to the aforementioned THI score and NRS scales. All other descriptive statistical analyses were performed using SPSS software (version 20.0, SPSS Inc., Chicago, IL). For all analyses, statistical significance was set at P < 0.05.

## Results

### Improvement in questionnaire scores 3 months after the initial TRT session

As summarized in Table 1, the mean THI scores improved significantly from the pre-TRT mean of 57.8 ± 20.7 to a 3 month-post-TRT mean of 40.4 ± 20.8 (*t* = 5.825, *P* < 0.001, paired *t*-test). In addition, NRS loudness (from 7.0 ± 2.0 to 5.6 ± 2.3, *t* = 3.525, *P* = 0.006, paired *t*-test), NRS distress (from 7.4 ± 2.2 to 5.3 ± 2.4, *t* = 3.096, *P* = 0.013, paired *t*-test), and NRS perception (from 84.1 ± 24.9% to 66.5 ± 31.9%, *P* = 0.033, paired *t*-test) showed statistically significant improvements 3 months after the initial TRT session compared with the pre-treatment scores.

Based on Jastreboff’s definition^5^, 13 of 32 subjects were good responders (a decrease of 20 THI points or more). Of these 13 subjects, 12 had bilaterally normal hearing while only one had bilateral mild hearing loss less than 30 dB. Eleven of 13 had pure tone tinnitus while the other 2 had narrow band noise-like tinnitus. Six of 13 had unilateral tinnitus while the other 7 presented with bilateral tinnitus.

### Improvement in the THI score: source-localized correlation and connectivity analyses

**Figure 1:**
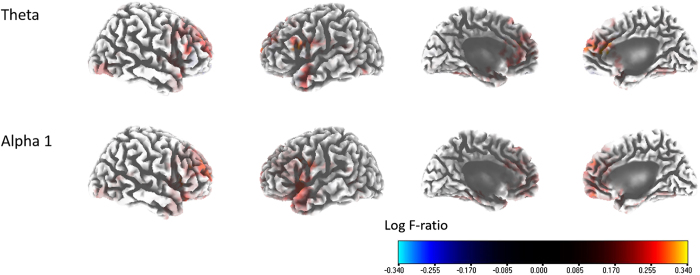
Source-localized correlation analysis between the percentage improvements in the Tinnitus Handicap Index (THI) score and the pre-tinnitus retraining treatment (TRT) resting-state quantitative electroencephalography data. The percentage improvements in the THI score were positively correlated with the activities of the left medial frontal cortex [Brodmann area (BA) 9], the left rostral anterior cingulate cortex (rACC; BA 24), and the right dorsolateral prefrontal cortex (DLPFC; BA 10) (i.e., the theta frequency band); and the activities of the the left insula (BA 13), the right DLPFC, the left rACC, the left pregenual anterior cingulate cortex (BA 32), and the left inferior frontal gyrus (BAs 45 and 47) (i.e., the alpha 1 frequency bands).

The percentage improvements in the THI scores were positively correlated with the activities of the left medial frontal cortex (mFC; BA 9), left rostral anterior cingulate cortex (rACC; BA 24), and right dorsolateral prefrontal cortex (DLPFC; BA 10) (i.e., the theta frequency band). The percentage improvements in the THI scores were also positively correlated with the activities of the left insula (BA 13), the right DLPFC, the left rACC, the left pregenual anterior cingulate cortex (pgACC; BA 32), and the left inferior frontal gyrus (IFG; BAs 45 and 47) (i.e., the alpha 1 frequency band; r = 0.640, P < 0.05) (Fig. 1). No significant correlations were apparent for the delta, alpha 2, beta 1, beta 2, beta 3, or gamma frequency bands. No clear correlations were evident between the percentage improvements in THI scores and connectivity among the 28 ROIs for the delta, theta, alpha 1, alpha 2, beta 1, beta 2, beta 3, or gamma frequency bands.

### Improvement in NRS loudness: source-localized correlation and connectivity analyses

**Figure 2:**
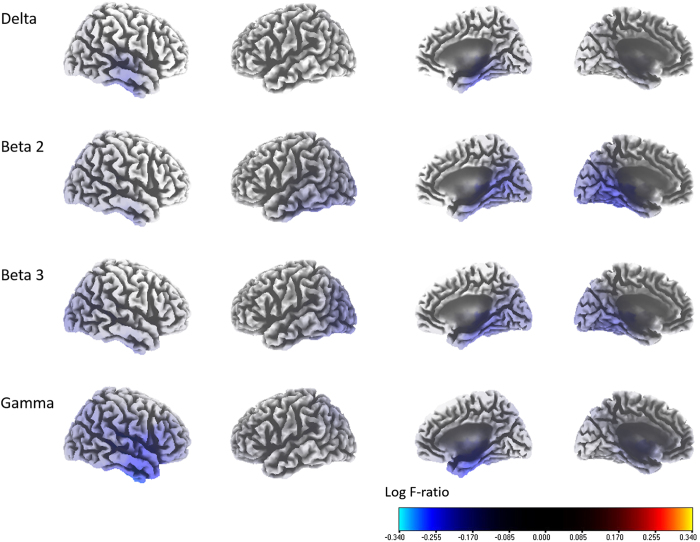
Source-localized correlation analysis between the percentage improvements in the numeric rating scale (NRS) of tinnitus loudness and the pre-TRT resting-state qEEG data. The activities of the right primary and secondary auditory cortices (BAs 41 and 21) (i.e., the delta and gamma frequency bands), and the activities of the parahippocampus (i.e., the delta, beta 2 and 3, and gamma frequency bands) increased with increasing THI scores.

The activities of the right primary and secondary auditory cortices (A1; BA 41, and A2; BA 21) (the delta and gamma frequency bands) and the parahippocampus (PHC) (the delta, beta 2 and 3, and gamma frequency bands) exhibited statistically significant negative correlations with the percentage improvements in NRS tinnitus loudness (r = −0.572, P < 0.05) (Fig. 2). No significant correlations were evident for the theta, alpha 1, or alpha 2 frequency bands.

**Figure 3:**
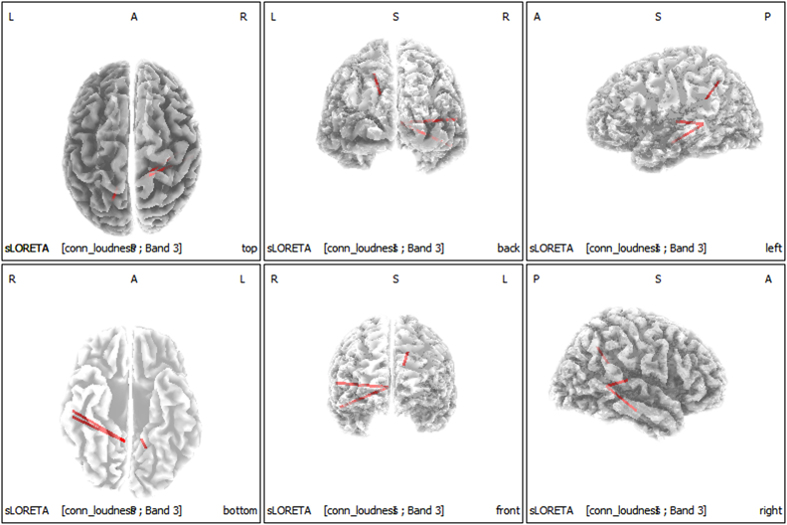
Connectivity comparisons between good and poor responders in the context of percentage improvements in the NRS of tinnitus loudness. The good responder group exhibited significantly better connectivity between the primary/secondary auditory cortices and the parahippocampus (i.e., the alpha 1 band) compared with the poor responder group.

No significant correlations were apparent between the percentage improvements in NRS tinnitus loudness and connectivity among the 28 ROIs. However, when we compared the connectivity between upper 16 responders and lower 16 responders using the median-split analysis in terms of percentage improvements in NRS tinnitus loudness, the upper 16 responders exhibited significantly better connectivity between the primary/secondary auditory cortices and the parahippocampus compared with the lower 16 responders (the alpha 1 frequency band) (Fig. 3).

### Improvement in NRS perception: source-localized correlation and connectivity analyses

**Figure 4:**
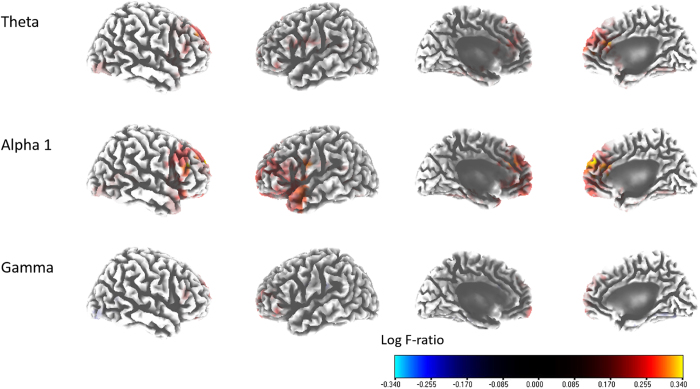
Source-localized correlation analysis between the percentage improvements in the NRS of tinnitus perception and the pre-TRT resting-state qEEG data. The percentage improvement in NRS tinnitus perception correlated positively with the pre-TRT localized activities of the rACC and the right DLPFC (BAs 9 and 10) (i.e., the theta frequency band); and the activities of the bilateral rACC, bilateral pregenual ACC, and right DLPFC (i.e., the alpha 1 band); and the activities of the left orbitofrontal cortex (BA 11) and right medial frontal cortex (i.e., the gamma band).

The percentage improvements in NRS tinnitus perception correlated positively with the pre-TRT source- localized activities of the right rACC and the right DLPFC (BAs 9 and 10) (i.e., the theta frequency band); those of the bilateral rACC, bilateral pgACC, and right DLPFC (i.e., the alpha 1 frequency band); and those of the left orbitofrontal cortex (OFC; BA 11) and right mFC (i.e., the gamma frequency band) (r = 0.865, P < 0.05) (Fig. 4). No significant correlations were evident for the delta, alpha 2, beta 1, beta 2, beta 3, or gamma frequency bands. The connectivity analyses showed no statistically significant correlations upon median-split group comparisons.

## Discussion

In the time since it was first introduced by Jastreboff^45^, TRT has become one of the most important treatment options for patients with chronic debilitating tinnitus. However, although nearly 20 years have passed since TRT was introduced, the exact mechanism of improvement is not fully understood, and no predictor of the degree of improvement after TRT is available. Moreover, to the best of our knowledge, although the neurophysiological model of tinnitus claims the involvement of cortical structures, such as the auditory cortex and limbic system, the neural substrates important in terms of treatment outcomes have never been investigated using functional neuroimaging modalities.

Therefore, the current study explored neural substrates that might predict short-term treatment outcomes after TRT; our findings are intriguing. The percentage improvements in the THI scores correlated positively with the activities of the left mFC, the left rACC, and the right DLPFC for the theta band; and those of the left insula, the right DLPFC, the left rACC/pgACC, and the left IFG, for the alpha 1 band. In terms of percentage improvements in tinnitus loudness, the activities of the right A1 and A2 (the delta and gamma frequency bands) and the PHC (the delta, beta 2 and 3, and gamma frequency bands) correlated negatively with such improvements. Additionally, the percentage improvements in NRS tinnitus perception correlated positively with the pre-TRT activities of the right rACC/DLPFC for the theta frequency band; those of the bilateral rACC, the bilateral pgACC, and the right DLPFC for the alpha 1 frequency band; and those of the left OFC and right mFC for the gamma frequency band.

### The autonomic nervous system controls tinnitus-related distress

The percentage improvements in the THI scores, reflecting the percentage improvements in tinnitus-related distress, correlated positively and significantly with the activities of the left mFC, the left rACC, and the right DLPFC for the theta frequency band; and those of the left insula, the right DLPFC, the left rACC/pgACC, and the left IFG, for the alpha 1 frequency band (Fig. 1). The classical neurophysiological model of tinnitus suggests that the emotional component of tinnitus is attributable to changes in the limbic system, which interacts with the autonomic nervous system^45^. In this regard, the current study, which revealed a positive correlation between the source-localized activity of the left insula and the extent of improvement in tinnitus-related distress, is in agreement with the classical neurophysiological model of tinnitus; the left insula is the center of parasympathetic tone control^46^,^47^. Thus, a relatively well-functioning left insula is a prerequisite for improvement in tinnitus-related distress in patients who undergo TRT. This is also in accordance with the findings of a previous study that showed a correlation between tinnitus-related distress and sympathetic activation, in part mediated via the right anterior insula^48^. In particular, the right insula activates the sympathetic nervous system and eventually increases tinnitus-related distress, but the left insula activates parasympathetic activity and thus decreases distress.

Together with the insula, other areas, such as the rACC and pgACC, are components of the “salience network” that influences the interoceptive-autonomic center^49^. One group recently developed an integrative model of tinnitus, suggesting that the salience network is the core modulator of tinnitus-related distress; the network controls the autonomic nervous system^17^. Our results also fit this theory; the activities of the rACC and pgACC were positively correlated with the percentage improvements in THI scores. Again, these results confirmed the classical neurophysiological model of tinnitus in terms of the importance of autonomic nervous system control in relieving tinnitus-related distress.

### A negative correlation between improvements in tinnitus loudness and the activities of core generators of tinnitus

The percentage improvements in tinnitus loudness were statistically significantly, but negatively, correlated with the activities of the right A1 and A2 for the delta and gamma frequency bands; and that of the PHC for the delta, beta 2 and 3, and gamma frequency bands (Fig. 2). Additionally, when we compared the connectivity between good and poor responder groups by performing median-split analysis of the percentage improvements in NRS tinnitus loudness, the good responder group exhibited significantly better functional connectivity between the primary/secondary auditory cortices and the PHC (the alpha frequency band) compared with the poor responder group (Fig. 3).

One recent report suggested that the auditory cortex supplies missing auditory information to tinnitus patients with minimal hearing loss, while the auditory memory stored in the PHC^50^ supplies missing information to patients with severe hearing loss^51^. More recently, a rs-qEEG study involving a large number of tinnitus patients showed that auditory cortical activity was increased in patients with little or no hearing loss compared with non-tinnitus controls, whereas relative increases in PHC activity were evident in patients with more severe hearing loss^52^. Moreover, another recent report showed that the subjective tinnitus loudness network consisted of the ACC/insula, the PHC, and the A1^53^. Judging from these recent findings, our results can be interpreted as follows: the stronger the tinnitus generators, the poorer the improvement in tinnitus loudness. That is, a relative increase in the activity of the A1 or PHC, the core generators of tinnitus and the core components of the loudness network, tends to preclude successful outcomes after TRT in terms of tinnitus loudness.

Our finding of significantly increased connectivity between the primary/secondary auditory cortices and the PHC in patients experiencing improvement in tinnitus loudness compared with those showing less improvement is in accordance with findings of a recent study, which showed that functional connectivity between the A1 and the PHC was enhanced in responders to intracranial A1 stimulation^54^. As hypothesized in the cited report, our results may indicate that the A1 is simply a functional entrance into a larger PHC-based tinnitus network; thus, patients with increased functional connectivity between A1 and PHC are more likely to enjoy improvements in tinnitus loudness, not only upon intracranial cortical stimulation but also after classical TRT. This suggests that the extent of the functional connectivity between the auditory cortex and the parahippocampus may serve as a biomarker for responsiveness to tinnitus treatment in general. If this is the case, it may be possible to develop new treatments.

### Importance of the rACC/pgACC-based noise canceling mechanism in decreasing tinnitus perception

The percentage improvement in the time during which tinnitus patients were aware of their tinnitus (i.e., NRS tinnitus perception) correlated positively with the pre-TRT activities of the right rACC/DLPFC (i.e., the theta frequency band) and those of the bilateral rACC, bilateral pgACC, and right DLPFC (i.e., the alpha 1 frequency band) (Fig. 4).

The rACC/pgACC is posited to be the core of the descending noise-canceling mechanism that counteracts peripheral auditory deafferentation-based pathological changes (e.g., tinnitus)^17^. In a recent proof-of-concept study correlating the daytime tinnitus awareness proportion with rs-qEEG data, the rACC/pgACC correlated negatively with tinnitus awareness; thus, these areas were suggested to be the core of the descending noise-cancellation system^9^. Based on such recent reports, our current study showing positive correlations between the percentage improvements in NRS tinnitus perception and pre-TRT rACC/pgACC activities is evidence of a causal relationship between these brain regions and tinnitus cancellation. In other words, a relatively well-functioning noise-cancelling core/mechanism is a prerequisite for improvement in tinnitus perception after TRT.

Further, right DLPFC activity correlated positively with the percentage improvements of both tinnitus-related distress and tinnitus perception. The classical model of Jastreboff’^4^ suggests that the DLPFC is a candidate for integration of the sensory and emotional aspects of tinnitus, and the DLPFC is indeed involved in the integration of emotion and cognition^55^. Therefore, the DLPFC may play roles in simultaneously abating both tinnitus-related distress and tinnitus perception.

### Strengths and limitations of this study

To the best of our knowledge, this is the first study to explore the cortical predictors of tinnitus improvement shortly after TRT. In this study, we found commonalities between the classical neurophysiological model and the newly suggested integrative model of TRT^4^,^17^. Therefore, as both models suggest, improvement in tinnitus loudness is related to the activity of tinnitus generators; the extent of tinnitus-related distress depends on the activity of the controller of the autonomic nervous system; and tinnitus perception relies on the activity of the noise-cancelling system. Additionally, the current study may be important in that our results serve as a milestone for development of precision medicine in the treatment of tinnitus. In other words, by predicting post-TRT outcomes using pre-treatment cortical oscillatory activities, patients who are expected to respond well to TRT may be recommended for TRT, while patients expected to show poor responses to TRT may be further counselled beforehand and perhaps be informed of other treatment options.

However, several limitations of the current study must be addressed. First, as we evaluated the treatment outcomes of TRT 3 months after the initial counselling session, the long-term effects of TRT should be re-evaluated in a follow-up study. Although previous studies indicated that highly significant improvements were evident after 3 months of treatment^6^,^56^, future studies correlating pre-treatment rs-qEEG data with long-term outcomes should be performed to reveal cortical predictors of the final TRT outcome. Second, information on the post-TRT status of our subjects is limited because only questionnaires were used for evaluation. In this regard, another follow-up study exploring post-TRT qEEG changes to reveal cortical changes after TRT, and to correlate changes in cortical activities with changes in subjective symptoms, should be performed. Third, we enrolled tinnitus subjects in relatively severe distress (i.e., a mean THI score of 57.8); few subjects were only mildly distressed. To explore the applicability of our current results to patients in less distress, a future study enrolling a greater number of subjects distressed to variable extents should be performed. Fourth, we have enrolled subjects with normal hearing or mild hearing loss and we did not take combined hyperacusis into account in the current study. As tinnitus patients with severe hearing loss have been reported to show different pattern of cortical activity compared to patients with no or mild hearing loss^57^. and tinnitus patients with combined hyperacusis have been reported to show different resting-state cortical activities from those without hyperacusis, these factors may affect outcome of TRT and the cortical predictors of TRT outcome may be changes if these factors are taken into account. Therefore, future studies exploring the role of the degree of hearing loss or combined hyperacusis in predicting TRT outcome should be performed.

## Conclusion

We revisited TRT with reference to cortical oscillation patterns measured by rs-qEEG. Our results underpin the role played by control of parasympathetic activity in improvement of tinnitus-related distress; the effects of A1/PHC-based tinnitus generators in the context of improving tinnitus loudness; and the role played by the descending noise-cancelling system in improving tinnitus perception. The current study supports both the classical neurophysiological model and the newly suggested integrative model of tinnitus, and our results serve as a milestone toward the development of precision medicine in the context of TRT.

## Additional Information

**How to cite this article**: Kim, S. H. *et al*. Neural substrates predicting short-term improvement of tinnitus loudness and distress after modified tinnitus retraining therapy. *Sci. Rep.* **6**, 29140; doi: 10.1038/srep29140 (2016).

## Figures and Tables

**Table 1:** Changes in the perceived tinnitus handicap scores three months after tinnitus retraining treatment.

	Pre-TRT	Post-TRT (3 months)	*P*-value
THI score	57.8 ± 20.7	40.4 ± 20.8	<0.001
NRS loudness	7.0 ± 2.0	5.6 ± 2.3	=0.006
NRS distress	7.4 ± 2.2	5.3 ± 2.4	=0.013
NRS perception (%)	84.1 ± 24.9	66.5 ± 31.9	=0.033

THI, tinnitus handicap inventory; NRS, numeric rating scale; TRT, tinnitus retraining treatment.
